# Mechanistic aspects of ameliorative effects of Eicosapentanoic acid ethyl ester on methotrexate-evoked testiculopathy in rats

**DOI:** 10.1007/s00210-023-02577-4

**Published:** 2023-07-14

**Authors:** Noha A. T. Abbas, Shaimaa S. El-Sayed, Samaa Salah Abd El-Fatah, Walaa M. Sarhan, Eman M. A. Abdelghany, Omnia Sarhan, Shireen S. Mahmoud

**Affiliations:** 1 Department of Clinical Pharmacology, Faculty of Medicine, Zagazig University, Zagazig City, 44519 Egypt; 2 Department of Pharmacology and Toxicology, Faculty of Pharmacy, Zagazig University, Zagazig City, Egypt; 3 Department of Human Anatomy and Embryology, Faculty of Medicine, Zagazig University, Zagazig City, Egypt; 4 Department of Medical Biochemistry and Molecular Biology, Faculty of Medicine, Zagazig University, Zagazig City, Egypt; 5 Wake Forest Institute of Regenerative Medicine (WFIRM), Winston-Salem, NC USA; 6 Department of Pharmaceutics, Faculty of Pharmacy, Badr University, Cairo, Egypt

**Keywords:** Methotrexate, Eicosapentaenoic acid ethyl ester, PPAR-γ, 8-OHdG, Testicular injury, Autophagy

## Abstract

Disrupted spermatogenesis and testicular injury are among the devastating outcomes of methotrexate. A major contributor to methotrexate-induced testiculopathy is oxidative damage which triggers apoptosis and altered autophagy responses. Eicosapentaenoic acid ethyl ester (EPA-E) is an antihyperlipidemic derivative of omega-3 fatty acids that exhibited affinity to peroxisome proliferator-activated receptor-γ (PPAR-γ) that possesses both antioxidant and autophagy modulating properties. This is an exploratory study aiming at assessing the effectiveness of EPA-E to alleviate testicular damage induced by methotrexate. The specific exploratory hypothesis of this experiment is: EPA-E administration for 1 week to methotrexate-treated rats reduces testicular damage compared to control rats. As a secondary outcome, we were interested in identifying the implicated mechanism that mediates the action of EPA-E. In adult male Wistar rats, testiculopathy was achieved by a single methotrexate injection (20 mg/kg, *ip*). Rats received vehicle, EPA-E (0.3 g/kg/day, *po*) alone or with selective PPAR-γ antagonist (bisphenol A diglycidyl ether, BADGE) at 30 mg/kg/day, *ip* for 1 week. EPA-E recuperated methotrexate-attenuated serum total testosterone while reduced testicular inflammation and oxidative stress, restoring superoxide dismutase (SOD) while reducing malondialdehyde (MDA) and 8–hydroxy–2′-deoxyguanosine (8-OHdG). Methotrexate-induced testicular apoptosis (caspase-3 and p53) was suppressed upon EPA-E treatment. Besides, EPA-E curbed methotrexate-induced abnormal autophagy by downregulating LC3A/B and beclin-1. Interestingly, BADGE-coadministration reversed EPA-E beneficial actions. Collectively, our findings suggest PPAR-γ role in EPA-E-mediated mitigation of methotrexate-evoked testiculopathy via suppression of oxidative stress, apoptosis, as well as abnormal autophagy. Furthermore, EPA-E could be used as a preventive therapy for some testiculopathies mediated by oxidative stress.

**Graphical Abstract:**

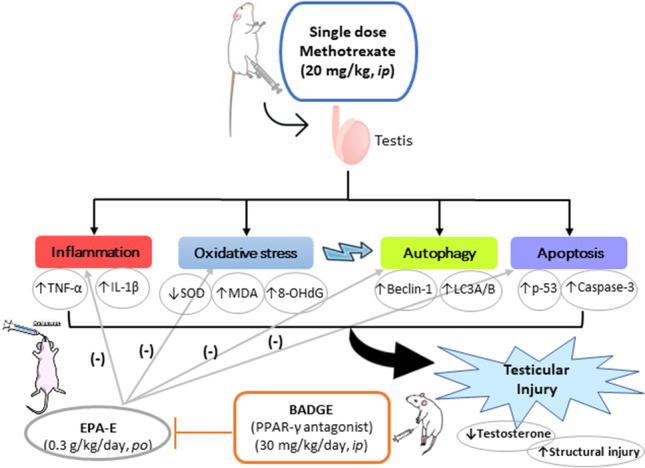

## Introduction

Methotrexate is a chemotherapeutic agent and disease-modifying antirheumatic drug (DMARD) that has been widely used in clinical practice. Methotrexate is a folic acid antagonist that interferes with nucleic acids synthesis (DNA and RNA) and has immunosuppressive effects; owing to these actions, it has been used in the treatment of malignancies and diverse auto-immune diseases such as rheumatoid arthritis and psoriasis (Elango et al. 2017). The cytotoxic actions of methotrexate are not limited to the diseased tissues, it extends to harm healthy ones, including hepatorenal (El-Sheikh et al. 2015) intestinal (El-Sheikh et al. 2016), cardiac (Al-Taher et al. 2020), and testicular tissues (Pinar et al. 2018; Wang et al. 2018). Even at a very low doses, methotrexate can provoke testiculopathy as manifested by testicular seminiferous tubules damage, sperm count reduction, and genetic mutations in sperms (Pinar et al. 2018; Wang et al. 2018; Wasilewski-Masker et al. 2014). Harmful effects of methotrexate are attributable, at least in part, to its induced reactive oxygen species (ROS) production that eventually causes testis and germ cells damage (Sherif et al. 2020). A predominant product of ROS-induced DNA oxidation is 8–hydroxy–2**′-**deoxyguanosine (8**-**OHdG) and considered as a crucial biomarker for oxidative burden and carcinogenesis (Valavanidis et al. 2009). Oxidative stress, in turn, can augment autophagy which further aggravates testicular injury; as abnormal autophagy suppresses Sertoli cells proliferation (Duan et al. 2016) while accumulated autophagosomes affect the integrity of blood-testis barrier (Yi et al. 2018), and subsequently leads to spermatogenesis disturbance and infertility. Thus, reducing oxidative stress-induced autophagy may be a useful target for prevention of drug induced male infertility. Furthermore, it has been reported that methotrexate adversely affects peroxisome proliferator-activated receptor-γ (PPAR-γ) (Mahmoud et al. 2017). PPAR-γ is a ligand-activated transcription factor that modulates lipid and glycemic homeostasis, induces anti-inflammatory cascade, and regulates expression of antioxidant genes (Girnun et al. 2002; Yu et al. 2002).

Eicosapentanoic acid ethyl ester (EPA-E) is a high-purity omega-3 fatty acid conjugate that was FDA-approved to reduce circulating triglyceride and the residual cardiovascular events (Nelson and Munger 2013). Eicosapentanoic acid exerts beneficial effect on inflammatory (Adkins and Kelley 2010) and oxidative (Mason and Jacob 2015) mechanisms implicated in atherosclerotic plaque development. Interestingly, omega-3 fatty acids have shown high affinity to PPAR-γ, and in breast cancer, they exert antiproliferative effects by modulating autophagy and apoptosis through PPAR-γ (Bonofiglio et al. 2016). This is an exploratory study aiming at assessing the effectiveness of EPA-E to alleviate testicular damage induced by methotrexate. The specific exploratory hypothesis of this experiment is: EPA-E administration for 1 week to methotrexate-treated rats reduces testicular damage compared to control rats. As a secondary outcome, we were interested in identifying the implicated mechanism that mediates the action of EPA-E. The current study is the first to demonstrate the preventive effect of EPA-E against methotrexate-induced testiculopathy highlighting its antioxidant potentials, especially on DNA-oxidation (8-OHdG) as well as its suppression to abnormal autophagy and this is the first aim in this study. The second aim was to scrutinize the implication of PPAR-γ in EPA-E-mediated effects.

## Materials and methods

### Drugs and vehicles

Methotrexate was obtained from Minapharm Pharmaceuticals (Cairo, Egypt) whereas EPA-E was obtained from Amarin Pharma Inc. (NJ, USA). Bisphenol A diglycidyl ether (BADGE) and dimethyl sulfoxide (DMSO) were purchased from (Sigma–Aldrich, Cairo, Egypt). BADGE was dissolved in 10% DMSO in phosphate buffered saline, PBS. All chemicals were of analytical grade.

### Animals

Our experiments were done on adult male Wistar rats (200 ± 20 g) purchased from the Faculty of Veterinary Medicine, Zagazig University, Egypt. Rats were then transferred to the animal facility at the Faculty of Medicine at Zagazig University, where they were housed in plastic cages with wood shave bedding (6 rats per cage) under standard humidity, temperature, and light/dark cycle (12/12), and receiving a standard diet and water *ad labitum*. All animal procedures were executed during the light period. The recommendations of the Weather all report and the National Institutes of Health guide for the care and use of laboratory animals were strictly followed.

### Methotrexate-induced testiculopathy experimental model

After 1 week of acclimatization, rats received methotrexate (single injection of 20 mg/kg, *ip*) according to (Sherif et al. 2020) to induce testiculopathy. For the control group, rats received equal volume of saline *ip*.

### Experimental protocol

Random assignment of twenty-four rats into 4 groups (6 rats per group) was performed as follows: group 1 (control): rats received vehicles (distilled H_2_O, *po*, and 10% DMSO in PBS, *ip*); group 2 to 4 comprised rats subjected to methotrexate injection and began to receive drugs or vehicles for 1 week starting on the day following methotrexate administration where group 2 (vehicle), group 3 (EPA-E); received EPA-E (0.3 g/kg/day, *po*) (Chen et al. 2020) and 10% DMSO in PBS *ip* daily; group 4 (EPA-E + BADGE); received EPA-E (0.3 g/kg/day, *po*) and BADGE (30 mg/kg/day, *ip*) (Li et al. 2016).

Number of animals selected according to pilot study under the supervision of IACUC members, according to 95% confidence interval and power calculation 80% and ratio of sample size 1:3 and mean of testicular of TNF-α of the control group was 16.4 ± 3 and mean of testicular TNF-α in EPA-E treated group was 21 ± 4 so the sample size will be 24 rats so 6 in each group.

### Blood and tissue sampling

At the conclusion and in heparinized tubes, blood samples were taken from retro-orbital plexus under sodium pentobarbital light anesthesia (50 mg/kg, *ip*) (Mohamed et al. 2020). Collected blood samples were centrifuged (3354 g, 15 min, 4 °C) and the aspirated serum was stored at -80 °C for later total testosterone measurement. Following exsanguination, euthanasia was assured by cervical dislocation. Scrotum was incised and testis were excised, rinsed then weighed. Left testis was homogenized in cold buffer composed of 50 mmol/L potassium phosphate (pH 7.4) and 1 mmol/L EDTA (5 ml buffer per gram tissue), testicular homogenate was then centrifuged at 4000 rpm for 15 min at 4 °C, supernatants were kept at − 80 °C for further biochemical analysis. Right testis was fixed in 4% paraformaldehyde in 0.1 M phosphate buffer overnight at 4 °C, then specimens were dehydrated in graded ethanol, embedded in paraffin for histopathological and immunohistochemical examination.

### Assessment of testicular damage

#### Biochemical assessment of experimental testiculopathy

To evaluate hypogonadism, serum total testosterone was measured using rat ELISA kit purchased from (CUSABIO Inc., Wuhan, China). In testicular homogenates, inflammatory status was assessed by measuring testicular proinflammatory markers, tumor necrosis-α and interleukin 1-β (TNF-α and IL-1β, respectively) utilizing MyBioSource rat ELISA kits (CA, USA). Moreover, oxidative status was evaluated by antioxidant superoxide dismutase (SOD) and lipid peroxidation marker (malondialdehyde, MDA) measurements using ready-made colorimetric kit obtained from Biodiagnostic (Cairo, Egypt), whereas testicular 8-OHdG, a product of DNA oxidation, was assessed using rat ELISA kit purchased from CUSABIO Inc. (Wuhan, China). For apoptosis, proapoptotic marker, caspase-3, was assessed using rat ELISA kit from MyBioSource (CA, USA). All assays were conducted following the manufacturer’s instructions.

#### Testicular weight and testicular weight/body weight determination

To further provide supporting evidence for the primary endpoint, which is testicular damage, testicular weight and its ratio to body weight were used in this study as sensitive indicators for chemically induced organ damage and were employed as secondary endpoints.

#### Histopathological assessment of experimental testiculopathy

For histopathological examination, 5-μm thick-sections were cut from paraffinized blocks of testis using a microtome, then deparaffinized in xylene, hydrated in graded ethanol, and stained with hematoxylin and eosin (El-Sayed et al. 2021). Sections were then examined under a light microscope (LEICA ICC50 W). From captured images and using Image j analysis software (Fiji image j; 1.51n, NIH, USA), height of the germinal epithelium and diameter of seminiferous tubules were determined. Furthermore, Johnsen’s testicular score system was applied to quantify spermatogenesis activity where a score from 1 (absence of germ cells) to 10 (maximum spermatogenesis activity) was given to each tubule (Johnston et al. 2001).

#### Immunohistochemical staining of testicular tissues for p53, LC3A/B and beclin-1

Four-micrometer thickness serial sections were sliced, dewaxed, rehydrated, immersed in citrate buffer (10 mM, pH 6.0) and heated (98 °C for 30 min). To block the endogenous peroxidase activity, cooled down-sections were then washed and treated with 3% H_2_O_2_/methanol for 15 min. To block nonspecific binding, sections were treated with 1% goat serum albumin for 10 min at room temperature as previously described (El-Sayed et al. 2021). Treated sections were incubated overnight at 4 °C with either p53 polyclonal primary antibody (#MA5-12,453, Invitrogen, Carlsbad, CA, USA) at dilution 1:100, as proapoptotic marker, or primary antibodies for LC3A/B (#12,741, Cell signaling Technology, 1:500) at dilution 1:500 or beclin-1 (#3738, Cell Signaling Technology, MA, USA) at dilution 1:200, as autophagy response markers. Later, sections were incubated with biotinylated secondary antibody and avidin–biotin complex (Vectastain® ABC-peroxidase kit, Vector Laboratories, Burlingame, CA, USA). 3,3-diaminobenzidine solution was utilized for color development. According to the previously described method (Varghese et al. 2014), image J software plugin and immunohistochemistry (IHC) profiler were used for calculation of the positive areas percentage (stained in brown), data gathered from three testis sections per animal.

The measurements were obtained in non-overlapping ten fields in slides of five different rats (randomly choosen) in each group at × 400 magnification.

The person responsible for capturing images for histological and immunohistochemical analysis was blinded about different study groups.

### Statistical analysis

GraphPad Prism, version 9.4.1 (681) (CA, USA) was employed for data analysis. Data are displayed in bar graphs which represent mean ± standard deviation of the mean (SD) for *n* = 6 rats/group. Shapiro–Wilk normality test Bartlett’s test were used to assure normal distribution and to check the homogeneity of variance, respectively. The one-way analysis of variance (one-way ANOVA) followed by post hoc Tukey test was employed for multiple comparisons. A significant difference was granted for values of *p* < 0.05.

## Results

### Effect on body and testicular weights

**Fig. 1:**
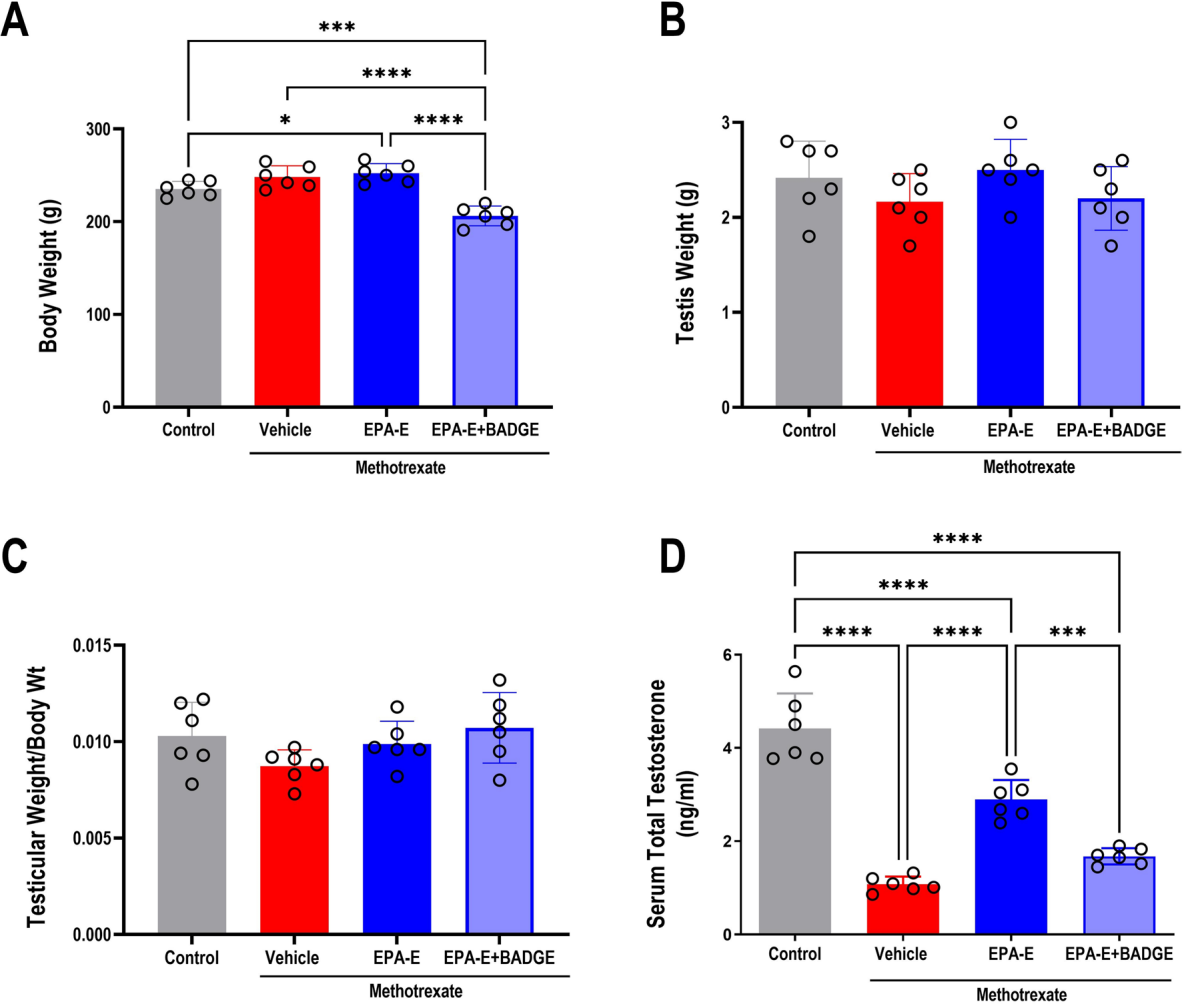
Effect 1-week treatment with Eicosapentanoic acid ethyl ester (EPA-E, 0.3 g/kg/day, *po*) alone or with bisphenol A diglycidyl ether (BADGE, 30 mg/kg/day, *ip*) on body weight (**A**), testicular weight (**B**), testicular to body weight ratio (**C**) and serum total testosterone level, following single methotrexate injection (20 mg/kg, *ip*). Values represent mean ± SD of different groups (*n* = 6/group). One-way ANOVA followed by Tukey’s test for multiple comparisons was used for analysis; ****p < 0.0001, ***p < 0.001, and **p < 0.01

Despite no significant differences in testicular weight (Fig. 1B) and relative testicular weight to body weight (Fig. 1C) among study groups, one-way ANOVA showed a significant main effect on body weight (*p* < 0.0001), where Tukey’s multiple comparisons revealed significant increase in EPA-E-treated methotrexate rats as compared to vehicle-treated ones (253.2 ± 3.280 vs 237.0 ± 3.786 g; *p* = 0.0406). Contrarily, cotreatment with BADGE significantly precipitated weight loss (207.2 ± 4.012 g) as compared to all other groups (*p* < 0.0001) (Fig. 1A).

### Effect on serum total testosterone levels

As depicted in Fig. 1D, one-way ANOVA showed a significant main effect on circulating total testosterone (*p* < 0.0001) where Tukey’s multiple comparisons revealed significant decline in its level in untreated methotrexate group (by 75.5%) compared to control (*p* < 0.0001). Despite the persistently lower serum testosterone in EPA-E-treated group compared to control (by 34.5%, *p* < 0.0001), on comparison with methotrexate group, EPA-E treatment resulted in significant 2.7 fold increase in serum testosterone (*p* < 0.0001). On the other hand, BADGE concomitant administration with EPA-E resulted in significant decrease in serum testosterone level, comparable to control (by 62.1%,* p* < 0.0001) and further when compared to EPA-E-treated group (by 42.1%, *p* < 0.0007).

### Effect on testicular histopathological alterations

**Fig. 2:**
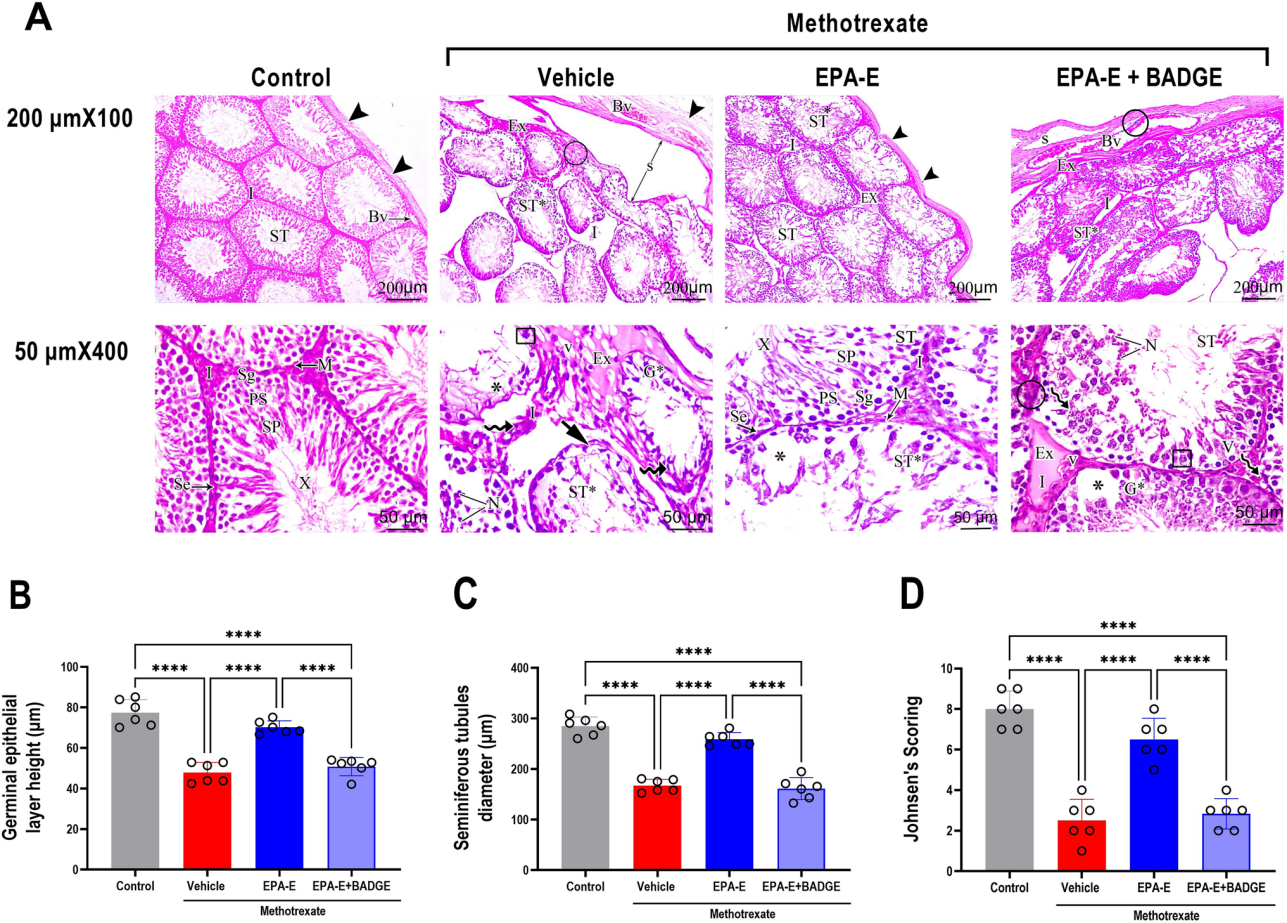
Effect 1-week treatment with Eicosapentanoic acid ethyl ester (EPA-E, 0.3 g/kg/day, *po*) alone or with bisphenol A diglycidyl ether (BADGE, 30 mg/kg/day, *ip*) following single methotrexate injection (20 mg/kg, *ip*) on testicular histopathology represented by photomicrograph of H&E-stained testicular sections (Scale bar, 200 μm × 100 and 50 μm × 400) from different study groups (**A**), germinal epithelial layer height (**B**), seminiferous tubule diameter (**C**) and Johnsen’s scoring (**D**). Seminiferous tubules (ST), thin capsule (arrow heads), blood vessel (BV), myoid cells (M), Sertoli cells (Se) spermatogonia (Sg), spermatocytes (PS), zone of spermatids (SP), spermatozoa (X), irregular tubules (ST*), capsular separation(S), interstitial spaces (I), Infiltration (within circle), exudate (Ex), multinucleated giant cells (in set), exfoliated epithelium in lumen (N), diminished layers of germinal epithelium(G*), irregular basement membrane (short arrow), atrophied cells (asterisks), apoptotic cells (zigzag arrows), and vacuolations (V). Values represent mean ± SD of different groups (*n* = 6/ group). One-way ANOVA followed by Tukey’s test for multiple comparisons was used for analysis; ****p < 0.0001

As displayed in Fig. 2A, features of methotrexate-induced testicular injury were found in the H&E-stained sections from methotrexate-rats, where methotrexate-group exhibited multiple irregular tubules with capsular separation and wide interstitial spaces in-between. Congested blood vessels, infiltration, and exudate within the capsule were seen as well as exfoliated epithelium in lumen and diminished layers of germinal epithelium. Furthermore, apoptotic cells were noticed in the tubules and the interstitial space. Contrarily, the control group exhibited normal histology manifested by seminiferous tubules that were mature functioning with complete spermatogenic series, tightly impacted and lined with thin capsule as well as narrow interstitial spaces. EPA-E-treatment nearly restored normal histological features demonstrated by normal capsule and narrow interstitium whereas seminiferous tubules retained their spermatogonia and stratified germinal epithelium; yet disordered seminiferous tubules displaying areas of atrophied germinal epithelium and in between tubules exudate were still seen. Coadministration of BADGE attenuated EPA-E-induced improvement where testicular sections from this group exhibited capsular separation, exudate, congested blood vessels within the capsule, irregular distribution of germinal epithelium, apoptotic cells in tubules’ periphery and in interstitial space, epithelium exfoliation in lumen and interstitial space vacuolation.

For further assessment of histopathological features, germinal epithelial layer height, seminiferous tubule diameter and Johnsen’s scoring were determined and presented in Fig. 2B, C, and D, respectively. One-way ANOVA showed a significant main effect (*p* < 0.0001) on germinal epithelial layer height, seminiferous tubule diameter and Johnsen’s scoring. Tukey’s multiple comparisons manifested significantly lower germinal epithelial layer height by 38.2%), seminiferous tubule diameter (by 41.3%) and worsened Johnsen’s scoring (by 68.8%) in methotrexate-group compared to control (*p* < 0.0001). EPA-E-treatment manifested significant increase in both germinal epithelial layer height and seminiferous tubule diameter (1.5 fold each) and augmented Johnsen’s score compared to methotrexate group (2.6 fold) (*p* < 0.0001). BADGE coadministration significantly reversed EPA-E effect, with 27.6, 37.7, and 56.4% reduction in germinal epithelial layer height, seminiferous tubule diameter and Johnsen’s scoring, respectively compared to EPA-E-treated and by 34.4, 43.3, and 64.6%, respectively, comparable to control (*p* < 0.0001).

### Effect on testicular proinflammatory markers

**Fig. 3:**
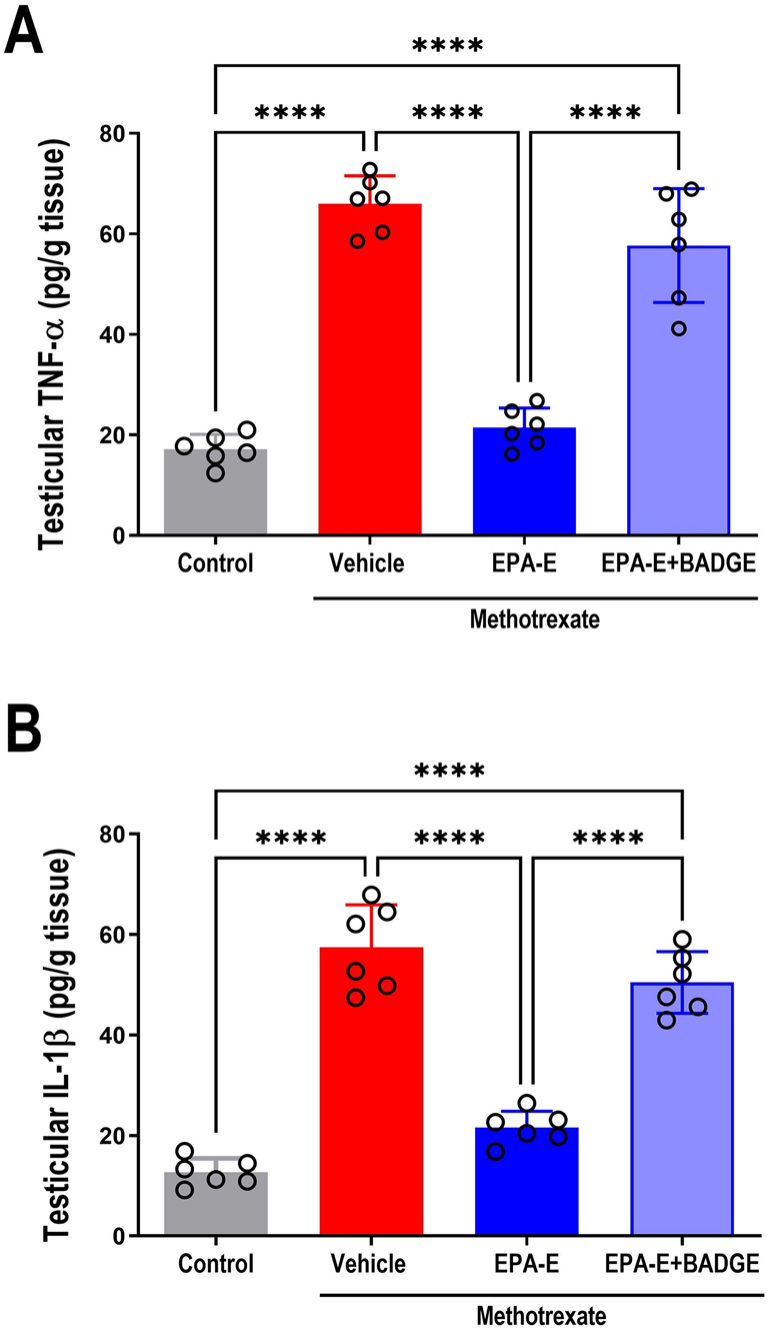
Effect 1-week treatment with Eicosapentanoic acid ethyl ester (EPA-E, 0.3 g/kg/day, *po*) alone or with bisphenol A diglycidyl ether (BADGE, 30 mg/kg/day, *ip*) on testicular levels of proinflammatory markers tumor necrosis factor-α (TNF-α, **A**) and interleukin 1-β (IL-1β, **B**) following single methotrexate injection (20 mg/kg, *ip*). Values represent mean ± SD of different groups (*n* = 6/group). One-way ANOVA followed by Tukey’s test for multiple comparisons was used for analysis; ****p < 0.0001

As depicted in Fig. 3, one-way ANOVA showed a significant main effect on testicular TNF-α and IL-1β (*p* < 0.0001) whereas Tukey’s multiple comparisons revealed significant augmented testicular inflammatory status (as manifested by 3.9 and 4.5 fold increase in TNF-α and IL-1β in methotrexate group compared to control (*p* < 0.0001). EPA-E successfully attenuated methotrexate-induced testicular inflammation by significant reductions of TNF-α and IL-1β (by 67.5 and 62.5%, respectively), compared to methotrexate-group (*p* < 0.0001). Whereas EPA-E effect was significantly attenuated upon coadministration of BADGE, exhibiting significant elevation in TNF-α and IL-1β (2.7 and 2.5 fold, respectively), compared to EPA-E-treated group and to control (*p* < 0.0001).

### Effect on testicular oxidative stress

**Fig. 4:**
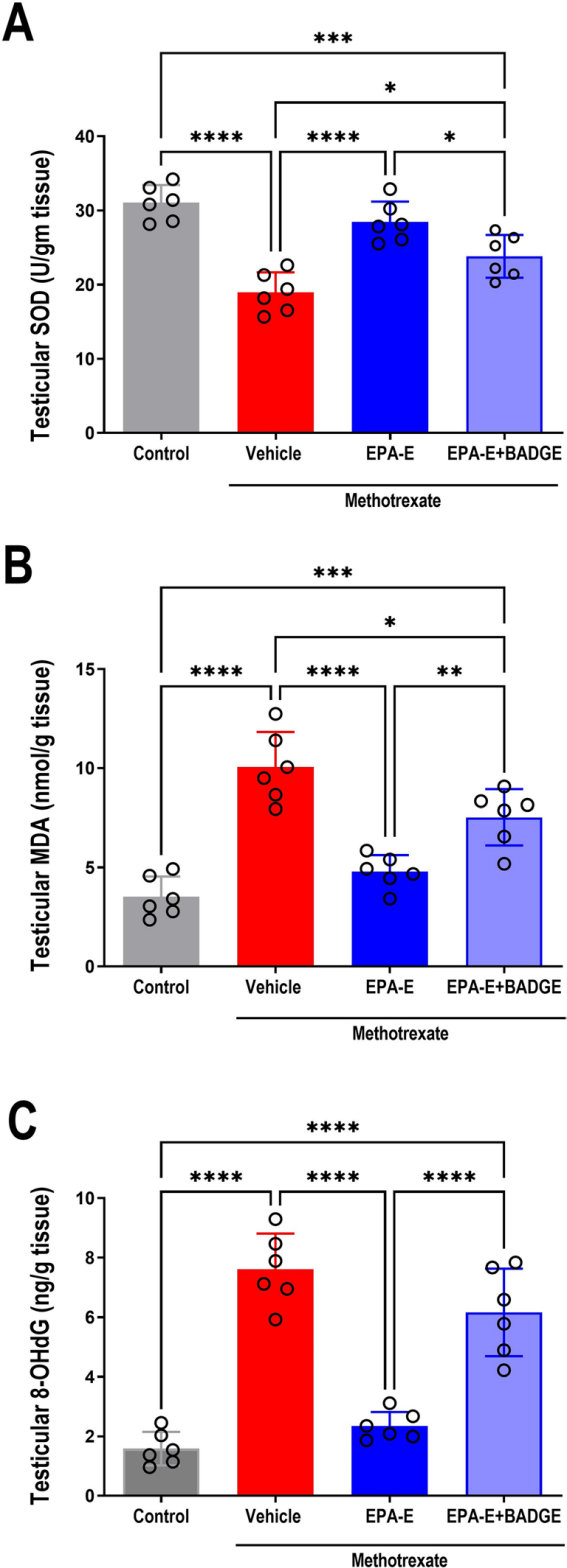
Effect 1-week treatment with Eicosapentanoic acid ethyl ester (EPA-E, 0.3 g/kg/day, *po*) alone or with bisphenol A diglycidyl ether (BADGE, 30 mg/kg/day, *ip*) on testicular oxidative stress demonstrated by superoxide dismutase (SOD, **A**), malondialdehyde (MDA, **B**) and 8–hydroxy**–**2**′-**deoxyguanosine (8**-**OHdG, **C**) following single methotrexate injection (20 mg/kg, *ip*). Values represent mean ± SD of different groups (*n* = 6/group). One-way ANOVA followed by Tukey’s test for multiple comparisons was used for analysis; ****p < 0.0001, ***p < 0.001, **p < 0. 01, and *p < 0.05

As a contributor to methotrexate-induced testicular damage, testicular oxidative stress was assessed by measuring antioxidant, SOD, lipid peroxidation product, MDA, and predominant DNA-oxidation product, 8**-**OHdG, as depicted in Fig. 4. One-way ANOVA demonstrated a significant main effect on testicular levels of SOD, MDA and 8**-**OHdG (*p* < 0.0001). Multiple comparisons with Tukey’s test revealed significant aggravated testicular oxidative stress in methotrexate group as manifested by significant decrease in SOD (by 38.9%) while augmentation in MDA (2.9 fold) and 8**-**OHdG (4.8 fold) comparable to control (*p* < 0.0001). Furthermore, in comparison with methotrexate-group, EPA-E alleviated oxidative stress exhibiting significant 1.5 fold rise in SOD while 52.3 and 69.2% reduction in MDA and 8**-**OHdG levels, respectively (*p* < 0.0001). BADGE coadministration with EPA-E exhibited a significant 23.3% reduction in SOD (*p* = 0.00008) and 2.1 fold increase in MDA (*p* = 0.0002), on comparison with control. Furthermore, compared to EPA-E-treatment, BADGE attenuated EPA-E-induced effects exhibiting significant reduction in SOD (16.3%, *p* = 0.0346) while significant increase in both MDA (1.6 fold, *p* = 0.0086) and 8**-**OHdG (2.6 fold, *p* < 0.0001).

### Effect on testicular apoptosis

**Fig. 5:**
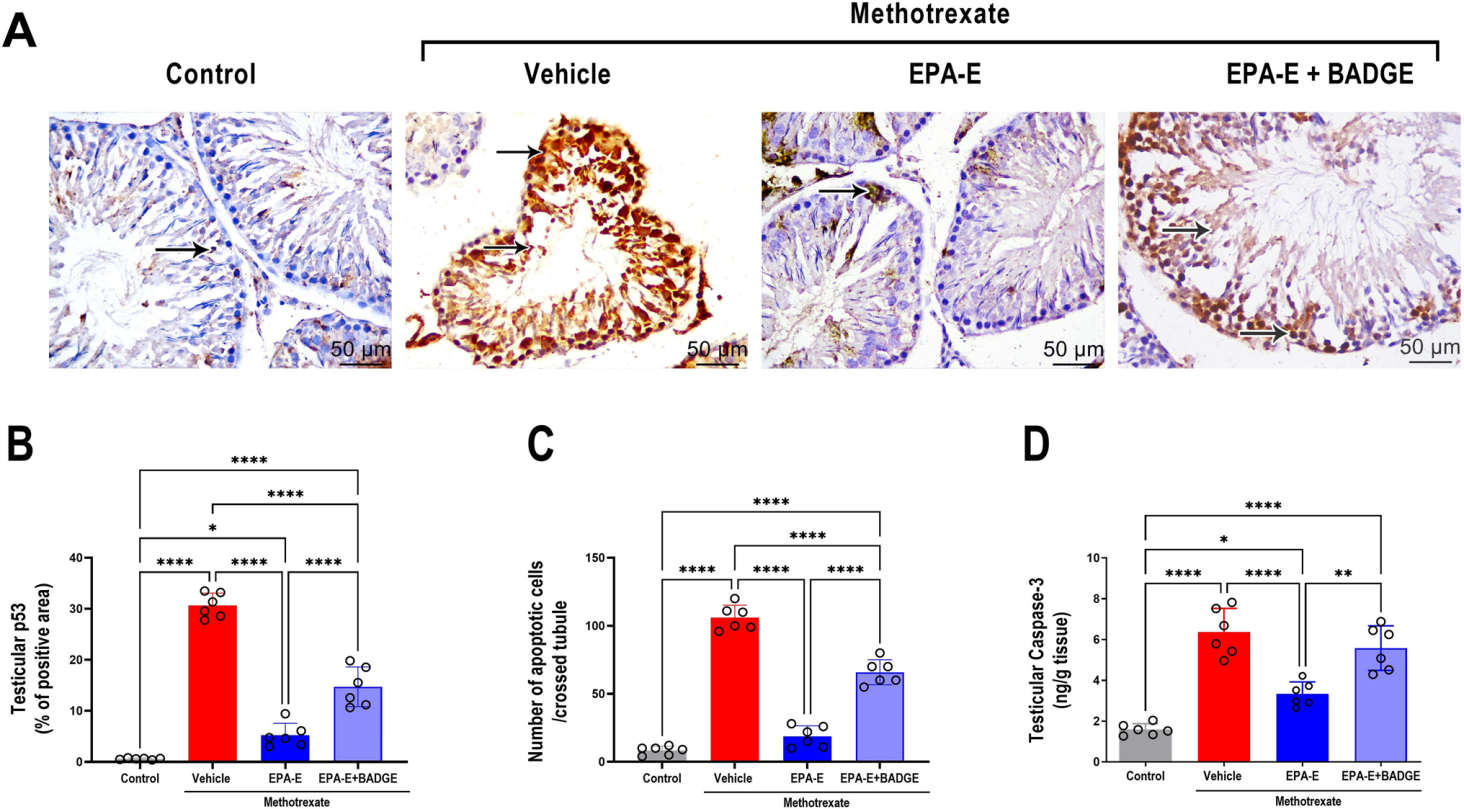
Effect 1-week treatment with Eicosapentanoic acid ethyl ester (EPA-E, 0.3 g/kg/day, *po*) alone or with bisphenol A diglycidyl ether (BADGE, 30 mg/kg/day, *ip*) following single methotrexate injection (20 mg/kg, *ip*) on testicular apoptosis demonstrated by immunohistochemical images of p53 expression (× 400 and scale bar, 50 μm) in different study groups (**A**), positive immune reaction for the target protein is demonstrated by the brown color and Arrows signifying dark brown staining of immunopositively stained nuclei of apoptotic cells. **B** is the quantification of p53 (% positive area) while panel **C** shows the number of p53-immunopositively stained nuclei. **D** shows testicular caspase-3. Values represent mean ± SD of different groups (*n* = 6/group). One-way ANOVA followed by Tukey’s test for multiple comparisons was used for analysis; ****p < 0.0001, ***p < 0.001, **p < 0. 01, and *p < 0.05

Immunohistochemical staining of testicular sections for proapoptotic marker (p53) and counting apoptotic cells per crossed tubule in the stained sections as well as measurement of testicular caspase-3 were used to assess methotrexate-induced testicular apoptosis as presented in Fig. 5. One-way ANOVA demonstrated a significant main effect on percentage of positive areas of p53-staining, number of apoptotic cells/crossed tubule and testicular caspase-3 (*p* < 0.0001). Tukey’s multiple comparisons revealed that methotrexate group exhibited significantly higher percentage of positive areas of p53-staining, increase in number of apoptotic cells per crossed tubule and augmented testicular levels of caspase-3 (49.2, 12.7, and 4 folds, respectively, *p* < 0.000), compared to control. EPA-E-treatment significantly attenuated apoptosis exhibiting lower percentage of positively stained areas of p53 and reduced in number of apoptotic cells per crossed tubule as well as testicular caspase-3 levels (83%, 82.4%, and 47.7%, respectively, *p* < 0.0001), compared to methotrexate group. BADGE coadministration with EPA-E significantly elevated percentage of positively stained areas of p53, number of apoptotic cells per crossed tubule and testicular caspase-3 levels as compared to control (23.7, 7.9, and 3.5 fold, respectively, *p* < 0.0001) or EPA-E-treated group (2.8, 3.5, and 1.7 fold, respectively, *p* < 0.001).

### Effect on autophagy

**Fig. 6:**
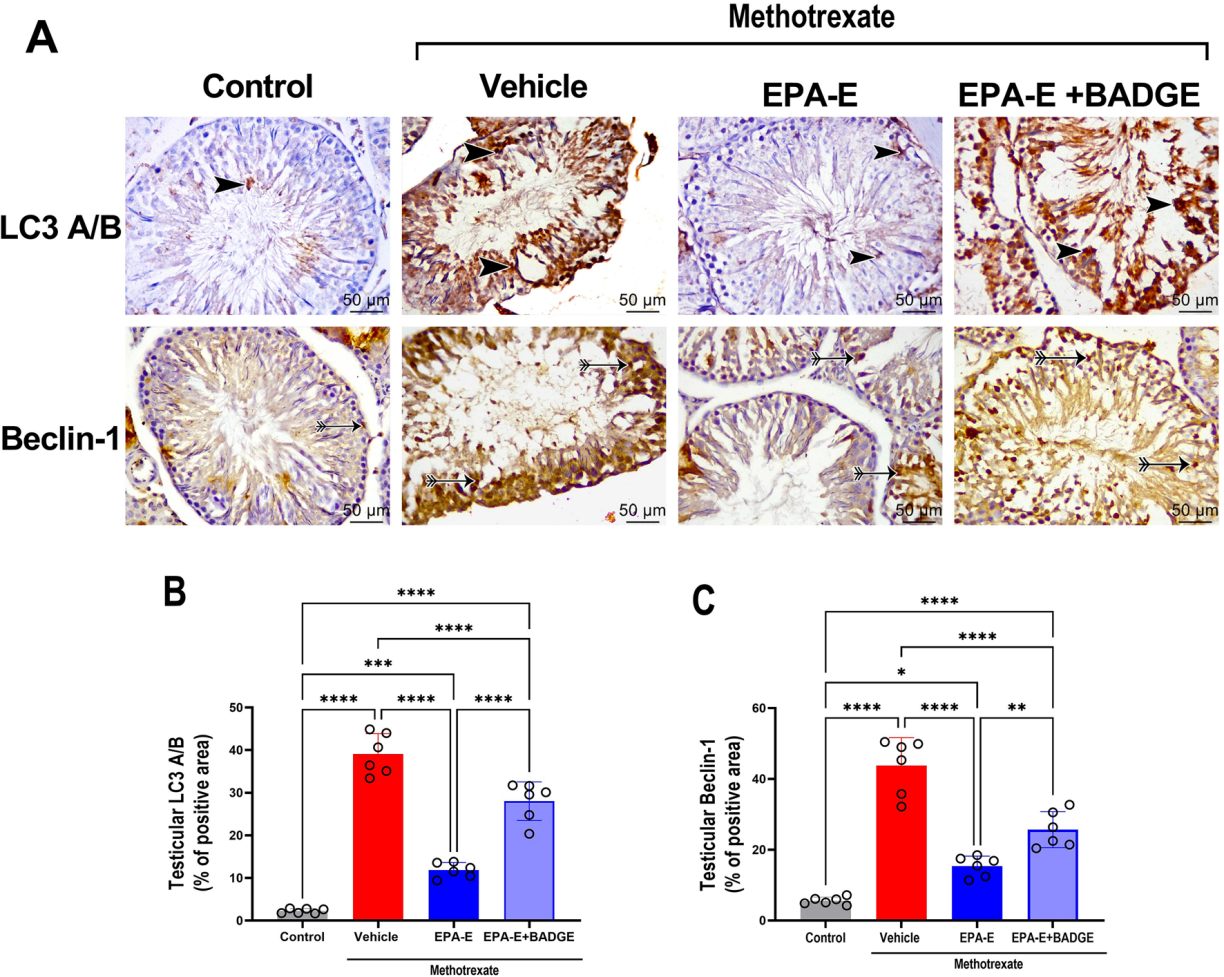
Effect 1-week treatment with Eicosapentanoic acid ethyl ester (EPA-E, 0.3 g/kg/day, *po*) alone or with bisphenol A diglycidyl ether (BADGE, 30 mg/kg/day, *ip*) following single methotrexate injection (20 mg/kg, *ip*) on changes in autophagy flux proteins in testicular tissue demonstrated by immunohistochemical staining images of LC3A/B and beclin-1 in different study groups (**A**). Positive immune reaction for the target protein is demonstrated by the brown color, arrow heads for LC3A/B and double tailed arrow for beclin-1 (X 400 and Scale bar, 50 μm). The lower panels are the quantification of LC3A/B (**B**) and beclin-1 (**C**). Values represent mean ± SD of different groups (*n* = 6/group). One-way ANOVA followed by Tukey’s test for multiple comparisons was used for analysis; ****p < 0.0001, ***p < 0.001, **p < 0. 01, and *p < 0.05

Immunohistochemical staining of testicular tissues for autophagosome proteins, LC3A/B, and beclin-1 was performed to assess autophagic flux as depicted in Fig. 6. One-way ANOVA demonstrated a significant main effect on percentage of positive areas of autophagosome proteins, LC3A/B and beclin-1 (*p* < 0.0001). Multiple comparisons with Tukey’s test revealed abnormal autophagic flux in methotrexate group depicted as significant increase in percentage of positive areas of both LC3A/B and beclin-1 (17.3 and 7.8 fold, respectively, *p* < 0.0001), compared to control. The group treated with EPA-E exhibited moderate immune reaction in both LC3A/B and beclin-1, yet it remains significantly lower compared to methotrexate group (by 69.7 and 64.9%, respectively, *p* < 0.0001). On the other hand, BADGE coadministration with EPA-E showed significantly higher immunoreactivity in both LC3A/B and beclin-1 compared to control (12.4 and 4.6 fold, respectively, *p* < 0.0001) as well as when compared to EPA-E -treated group (2.4 fold, *p* < 0.0001 and 1.7 fold, *p* = 0.0084, respectively).

## Discussion

The maintenance of male fertility against adverse effects consequent to drug use is of great concern. A highly effective and widely used DMARD and chemotherapeutic agent, methotrexate, has been reported for its multiorgan toxicity including testis (Pinar et al. 2018)**.** It induces seminiferous tubules degeneration, reduces sperm count and causes sperm DNA damage (Pinar et al. 2018; Wang et al. 2018; Wasilewski-Masker et al. 2014)**.** EPA-E is a well-known antihyperlipidemic omega-3 fatty acid derivative that possesses anti-inflammatory and antioxidant actions (Adkins and Kelley 2010; Mason and Jacob 2015). Omega-3 fatty acids showed affinity to PPAR-γ and they exert antiproliferative effects by modulating autophagy and apoptosis through PPAR-γ as in breast cancer (Bonofiglio et al. 2016). As far as we know, this study is the first to demonstrate EPA-E-mediated amelioration of methotrexate-induced testiculopathy and to investigate whether or not PPAR- γ activation is implicated in EPA-E-conferred effects.

Herein, methotrexate-induced testicular dysfunction was demonstrated by significant decline in serum total testosterone levels that was restored upon EPA-E treatment. The decreased testosterone level with methotrexate administration may be attributable to increased ROS in Leydig cells precipitating testosterone synthesis disorder (Reddy et al. 2004). On histological level, methotrexate-induced testicular injury characterized by seminiferous tubule degeneration with observed multinucleated giant cells (a cluster of degenerated germ cells) which is consistent with a previous report (Vardi et al. 2009). In the present study, widening of the intercellular spaces was observed in methotrexate group which may be attributable to blood–testis barrier disruption upon exposure to ROS, permitting entrance of excess water and toxic agents between the spermatogenic cells, and eventual widening of the intercellular spaces. In addition, exfoliation of the spermatogenic cells into seminiferous tubules lumen that was observed in methotrexate group herein may be attributed to Sertoli cells’ cellular processes destruction (Naghdi et al. 2016). Moreover, methotrexate caused significant decrease in the diameter of seminiferous tubules, the height of germinal epithelium and spermatogenic cell content estimated by Johnsen’s scoring which is consistent with a previous study reporting similar findings (Padmanabhan et al. 2009). Methotrexate-induced damage to testicular structures and germ cells may be explained by methotrexate-induced generation of ROS (Phillips et al. 2003). EPA-E-treatment restored most of normal histological features.

Moreover, in the current study, methotrexate administration significantly increased testicular levels of proinflammatory markers (TNF-α and IL-1β) which is in harmony with previous studies (Morsy et al. 2020; Sherif et al. 2020). Both TNF-α and IL-1β activate the inflammatory cascade by stimulating the production of several chemokines and cytokines, adhesion molecules and nitric oxide contributing to testicular injury (Lampiao and du Plessis 2008). Reduction of testicular IL-1β and TNF-α levels upon EPA-E treatment indicates its conferred anti-inflammatory actions in abating methotrexate-induced testicular toxicity. These findings are in alignment with previous studies that demonstrated direct anti-inflammatory properties of EPA-E as in acute pancreatitis (Berger et al. 2020), type II diabetes (Brinton et al. 2013) and COVID-19 patients (Kosmopoulos et al. 2021).

A major contributor to methotrexate-induced testicular injury is oxidative stress resulting from imbalance between ROS generation and ROS scavenging system (Belhan et al. 2019; Yuluğ et al. 2013). Testis are labile to oxidative stress-induced injury mostly due to its high content of polyunsaturated fatty acids and its low antioxidant capacity (Vernet et al. 2004). The current findings demonstrated reduction in antioxidant defense (SOD) while augmentation in lipid peroxidation product (MDA) in methotrexate group. On the other hand, EPA-E treatment increased SOD level while attenuated MDA elevation, which might be explained, at least in part, by free radical scavenging activity and alleviation of testicular lipids (Olutope et al. 2014). Several studies reported antioxidant activity of EPA-E as in myocardial infarction (Chen et al. 2020) and hypertriglyceridemia (Sherratt and Mason 2018). Generated ROS can damage lipids, proteins as well as DNA, ROS attacks DNA strands and oxidizes guanosine resulting in the formation of 8**-**OHdG which is used as a marker for the severity of DNA oxidative damage (Nakae et al. 2000); therefore, possible protective effect of EPA-E was evaluated by assessing 8**-**OHdG levels in testicular tissue. Herein, 8**-**OHdG was significantly elevated in methotrexate group, while significantly reduced upon EPA-E treatment suggesting reduced DNA damage.

Furthermore, mitochondrial apoptotic cell death is stimulated by disrupted Bax/Bcl-2 ratio and later activation of caspase enzymes that are mainly activated by ROS generation (Kucukler et al. 2020). Caspase-3 is the most crucial caspase enzyme and its level is a hallmark of apoptosis (Owumi et al. 2019)**.** In the current study, methotrexate increased testicular caspase-3 suggesting activation of testicular apoptosis. These findings are further supported by immunohistochemical staining findings for proapoptotic p53, where methotrexate group exhibited significant increase in testicular p53 as well as greater number of apoptotic nuclei. Furthermore, the current findings agreed with previous studies on methotrexate-evoked testiculopathy in which similar findings were reported (Morsy et al. 2020; Sherif et al. 2020; Yuluğ et al. 2013). On EPA-E treatment, testicular caspase-3, p53 as well as number of apoptotic nuclei were significantly attenuated suggesting potential anti-apoptotic properties of EPA-E which is consistent with a previous study that reported antiapoptotic effect of eicosapentaenoic acid in palmitic acid-induced endothelial dysfunction (Lee et al. 2014).

Another important mechanism in cellular homeostasis is autophagy. Autophagy role in testicular injury is divergent where moderate autophagy protects against testicular damage as in hyperglycemia (Sato et al. 2016) or hypoxia (Zhang et al. 2016) while abnormal autophagy impairs spermatogenesis and predisposes to infertility (Duan et al. 2016). Abnormal autophagy is induced by augmented ROS (Duan et al. 2016) which is similar to what happens with methotrexate. In the current study, immunohistochemical staining for autophagic markers and LC3A/B and Beclin-1 in testicular sections demonstrated augmented autophagic activity in methotrexate group which further contributed to testicular damage. A previous study suggested that methotrexate treatment can develop resistance through autophagy induction (Xu et al. 2015). Another in vitro study on spermatocyte cell line (GC2) suggested that ROS signaling mediates methotrexate-induced autophagy and apoptosis which is consistent with our findings. On the other hand, EPA-E treatment alleviated methotrexate induced autophagy via reducing the expression of both LC3A/B and Beclin-1 which can be explained by its reported antioxidant activity (Chen et al. 2020; Sherratt and Mason 2018).

Differentiating germ and Sertoli cells highly express PPAR-γ which plays a pivotal role in spermatogenesis and sperm capacitation; thus it constitutes a possible target for male infertility therapy (Thomas et al. 2011). As reported in previous studies, PPAR-γ ameliorates testicular injury via alleviating oxidative stress, suppressing inflammation and disrupting germ cell apoptosis as in diabetes (El-Twab et al. 2016) or gentamycin-induced testicular injury (El-Sayed et al. 2022). In the present work, EPA-E successfully attenuated methotrexate induced testicular damage as it restored testosterone levels and normal histological features while suppressed inflammation, oxidative stress, apoptosis, and abnormal autophagy. In an attempt to scrutinize the implication of PPAR-γ in ameliorative effects conveyed by EPA-E, a selective PPAR-γ antagonist (BADGE) was co-administered with EPA-E. BADGE attenuated EPA-E-mediated testicular protection as demonstrated by declined testosterone, deteriorated histology, augmented inflammatory status as well as oxidative stress, besides increased apoptosis, and autophagy.

In Conclusion, the ability of EPA-E to mitigate methotrexate-induced testiculopathy may be attributable to its anti-inflammatory, antioxidant and antiapoptotic effects. The current study gives an insight on EPA-E-suppression of DNA oxidative damage (8**-**OHdG) and abnormal autophagy. Furthermore, the current findings strongly suggest the role of PPAR-γ receptors in EPA-E-mediated prevention of methotrexate-induced testiculopathy. Collectively, EPA-E, being a PPAR-γ agonist, could be used as adjuvant therapy with methotrexate to counteract its induced testicular injury.

### Study limitation

We counted on serum testosterone level as a measure of testicular function. It would have been better to assess sperm count and motility. This is considered as a limitation of our study; therefore, it will be considered in our future studies.

## Data Availability

The data generated and analyzed during this study will be available from the corresponding author upon reasonable request.

## Abbreviations

EPA-E: Eicosapentaenoic acid ethyl ester
PPAR-γ: Peroxisome proliferator-activated receptor-γ
DMARD: Disease modifying antirheumatic drug
BADGE: Bisphenol A diglycidyl ether
SOD: Superoxide dismutase
8-OHdG: 8–Hydroxy–2′-deoxyguanosine
MDA: Malondialdehyde
ROS: Reactive oxygen species
